# Creating a 3D microbial and chemical snapshot of a human habitat

**DOI:** 10.1038/s41598-018-21541-4

**Published:** 2018-02-27

**Authors:** Clifford A. Kapono, James T. Morton, Amina Bouslimani, Alexey V. Melnik, Kayla Orlinsky, Tal Luzzatto Knaan, Neha Garg, Yoshiki Vázquez-Baeza, Ivan Protsyuk, Stefan Janssen, Qiyun Zhu, Theodore Alexandrov, Larry Smarr, Rob Knight, Pieter C. Dorrestein

**Affiliations:** 1 Department of Chemistry, University of California San Diego, La Jolla, CA USA; 2 Collaborative Mass Spectrometry Innovation Center, Skaggs School of Pharmacy and Pharmaceutical Sciences, University of California at San Diego, La Jolla, CA USA; 3 Department of Computer of Science and Engineering, University of California San Diego, La Jolla, CA USA; 4 Structural and Computational Biology Unit, European Molecular Biology Laboratory, 69117 Heidelberg, Germany; 5 California Institute for Telecommunications and Information Technology, University of California San Diego, La Jolla, CA USA; 6 Center for Microbiome Innovation, University of California San Diego, La Jolla, CA USA; 7 Department of Pediatrics, University of California San Diego, La Jolla, CA USA

## Abstract

One of the goals of forensic science is to identify individuals and their lifestyle by analyzing the trace signatures left behind in built environments. Here, microbiome and metabolomic methods were used to see how its occupants used an office and to also gain insights into the lifestyle characteristics such as diet, medications, and personal care products of the occupants. 3D molecular cartography, a molecular visualization technology, was used in combination with mass spectrometry and microbial inventories to highlight human-environmental interactions. Molecular signatures were correlated with the individuals as well as their interactions with this indoor environment. There are person-specific chemical and microbial signatures associated with this environment that directly relate who had touched objects such as computers, computer mice, cell phones, desk phone, table or desks. By combining molecular and microbial investigation forensic strategies, this study offers novel insights to investigators who value the reconstructing of human lifestyle and characterization of human environmental interaction.

## Introduction

Humans constantly interact with their environment and unintentionally leave behind a plethora of chemical and microbial traces^1,2^. Many of these signatures are substantially dictated by their habits and lifestyle choices of the individual^3–5^. It was hypothesized that by studying molecular and microbial clues across an office space, associations between objects of that environment and its inhabitants can be made. Since it may even be possible to identify personal habits for each individual from these signatures^4^, such trace evidence could tremendously benefit forensic science^6^. More importantly, it is necessary to properly document and understand how to best utilize such information. Solving this task has the potential to modernize forensic studies especially when correlating a particular individual to a surface or object within an environment is paramount to ‘solving a case’. Additionally, beyond forensics applications, this investigation also addresses the growing interest in understanding the role of humans as unique reservoirs of chemicals and how their microbes contribute to the overall molecular diversity of a built environment^6^.

To analyze the molecular and microbial traces of humans in a built environment, we employed surface sampling and visualized these findings with 3D molecular cartography^1,4^. In 3D molecular cartography, samples are collected across a space and the data is then mapped using their coordinates of the 3D model. The 3D model of the office with volunteers sitting at the conference desk was created using a 3D scanner (Supplemental Fig. 1). A detailed analysis of uniquely distributed molecules and microbes provided interesting clues into the lifestyles of each volunteer as previously demonstrated to individuals and their phones^4^. Using Global Natural Products Molecular Social networking^7^ (GNPS), molecular networking was able to correlate office molecules and office object molecules with volunteer’s person or personal care products. 16S rRNA amplicon sequencing was used to inventory bacteria taxonomy across volunteers and the office. 16S rRNA amplicon sequencing provides insights into the bacteria that are present in a sample. Liquid chromatography mass spectrometry is an analytical technique that separates and detects molecules based on their hydrophobicity and atomic weight. LC-MS/MS was used to inventory the molecules in the room. Such associations provide insights into the lifestyles of volunteers in the built environment. We further demonstrated that partial least squares analysis^8^ (PLS) can be used to distinguish volunteers’ skin metabolome and skin microbiome with each other and also with the biomes of the built environment. Lastly, we demonstrate that SourceTracker V.1^9^ can be used to identify the relationships between individuals and the overall molecular and microbial profiles observed in the environment.

## Results

The office environment included a desk, bookshelf, conference table, four chairs, garbage and recycling cans, the carpet-covered floor, phones, computers and computer accessories and walls. Four individuals inhabited the office environment for a one-time sample collection and were labeled as volunteers 1–4 (Supplemental Fig. 1). Volunteer 3 is the main inhabitant of the office; volunteer 1 had only been in the office twice in the past two years while volunteers 2 and 4 frequented the office 2–4 times a month for short periods (minutes to hours) of time during each visit. A total of 390 sites were sampled from the office environment; 276 sites were sampled on the skin, clothes, and accessories of volunteers. Each volunteer was sampled 33 times throughout his or her head, face, arms, legs, chest, and hand. Each built environment site was sampled between 2–17 times. To obtain both chemical and microbial inventories, each sample site was sampled twice within 1 cm apart making sure not to overlap extraction points. One swab from each sample site was subjected to untargeted liquid chromatography tandem mass spectrometry (LC-MS/MS) for detection of small molecules and the other swab was subjected to 16S rRNA amplicon sequencing for assessing the microbial taxa. The resulting data was overlaid onto the 3D model in the ‘ili web application^10^. No particular order was assigned during sample collection. The volunteers also provided 27 personal care products such as shampoo, toothpaste, shaving cream, sinus medication, deodorant, body wash, aftershave and essential oils for metabolomic analysis which later aided in assigning ownership of molecules across the molecular space.

**Figure 1:**
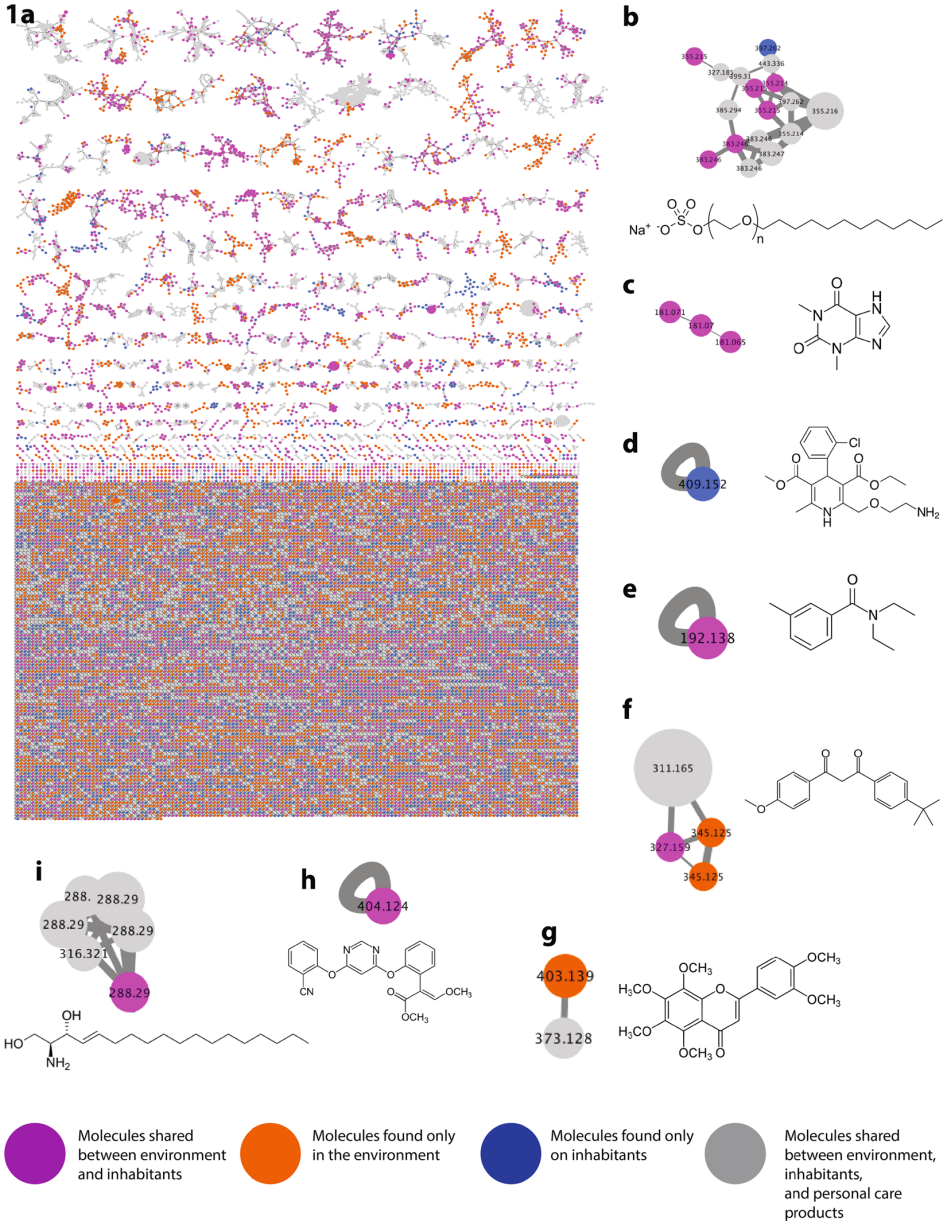
A molecular network of the office including its inhabitants and personal care products. The colors of the nodes illustrate that molecules are found on either the volunteers, built environment, or from personal care products of volunteers. (**a**) Blue nodes represent molecules exclusively found on volunteers. Orange nodes represent molecules exclusively found on environment. Pink nodes represent molecules shared between people and environment. Grey nodes represent molecules between environment, inhabitants and personal care products. (**b**) Sodium Lauryl Sulfate, *m/z* 397.262. (**c**) Theophylline *m/z* 181.071. (**d**) Amlodipine medication, *m/z* 409.152 (**e**) DEET, *m/z* 192.138 (**f**) Avobenzone, *m/z* 311.165 (**g**) Nobiletin, *m/z* 403.139 (**h**) Azoxystrobin, *m/z* 404.124 (**i**) D-erythro-sphingosine, *m/z* 288.29.

After LC-MS/MS data acquisition was performed, each sample site yielded thousands of raw spectral data points that when combined in GNPS and visualized in Cytoscape, produced a molecular network that described the chemistry of the office (Fig. 1a). A molecular network provides a visual representation of the molecules that were fragmented by tandem mass spectrometry^11^. After all sample sites were subjected to LC-MS/MS metabolomic profiling, a total of 301,230 raw spectral files were obtained. In an attempt to reduce low quality spectra, specific GNPS data analysis workflow parameters were used (min matched peak = 6, where the peaks are the ions in the spectrum after tandem mass spectrometry was performed, min cluster size = 2). During the construction of the molecular network, 21,732 spectra of the obtained 301,230 spectra were used to generate a molecular network of the office (Fig. 1a). Each node represents a unique molecule, while the edges connecting each node suggests molecular similarity. Self-looping nodes (single nodes and edges connecting to themselves), illustrate molecular uniqueness under the GNPS processing parameters. Within the network, 799 nodes (~4%), were matched to the reference spectra that make up the GNPS library. Of these nodes, 15,274 (70%) nodes were unique to the built environment (Fig. 1a). Volunteer 1, 2, 3, and 4 produced 5,903 (27.2%), 5,859 (26.9%), 5,390 (24.8%), and 5874 (27%) independent nodes respectively. The built environment shared 4,325 (19.9%), 4,154 (19.1%), 3,841 (17.6%), and 4,018 (18.4%) nodes with volunteers 1, 2, 3 and 4 respectively. 5,510 nodes were associated with personal product standards alone. Across the entire molecular network, 666 (3.1%) nodes were shared among all four volunteers, the built environment and volunteer personal care products. Figure 1g reveals representative molecules that were detected and annotated. It was also shown that each molecule was found to help with forensic analysis. Sodium lauryl sulfate (Fig. 1b), avobenzone (Fig. 1f), and D-erythro-sphingosine are personal care molecules shared between the inhabitants and the built environment. Theophylline (Supplemental Fig. 2c), DEET (Fig. 1e), and azoxystrobin (Fig. 1h) were molecules shared between the inhabitants and the environment. Amlodipine (Fig. 1d) was only found on the volunteers, while nobiletin (Fig. 1g) was found exclusively in the built environment

### Mapping the location of microbes and molecules

**Figure 2:**
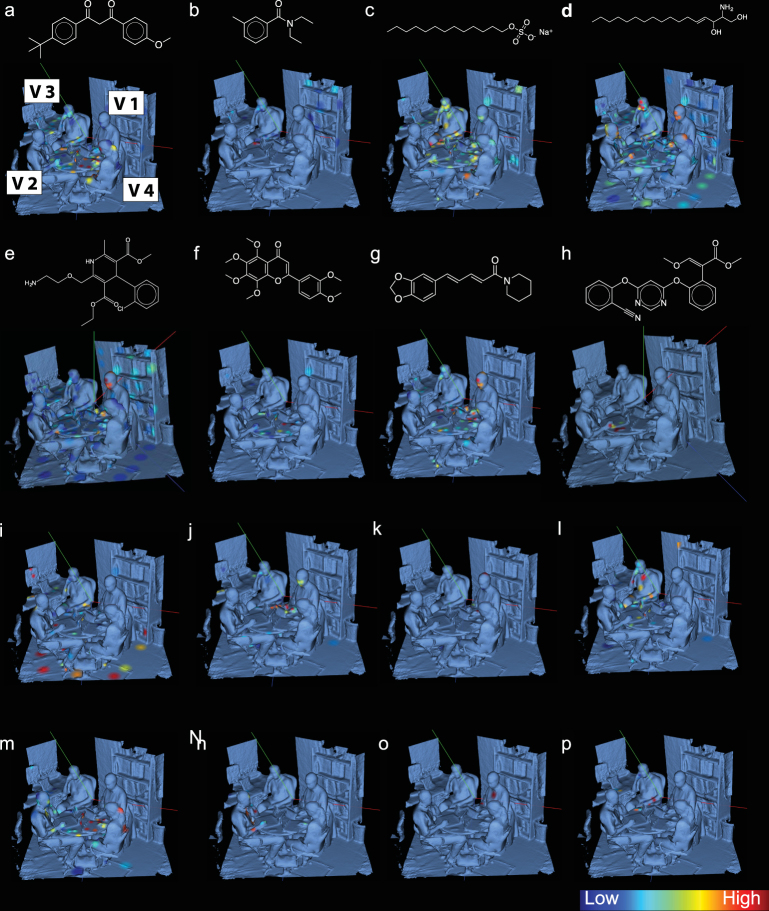
Distribution of molecules and microbial taxa in a human habitat and its volunteers 1–4. The MS data of the office environment sampling locations and relationships to the microbes in those same locations. (**a**) Avobenzone, (**b**) DEET, (**c**) Sodium Laureth Sulfate, (**d**) C17 Sphingosine, (**e**) Amlodipine (Norvasc), (**f**) Nobiletin, (**g**) Theophylline, (**h**) Azoxystrobin, (**i**) *Nocardiaceae*, (**j**) *Acinetobacter guillouiae*, (**k**) *Rhizobiales*, (**l**) *Synechococcus*, (**m**) *Actinomycetales*, (**n**) *Staphylococcus*, (**o**) *Veillonella parvula*, (**p**) *Chitinophagaceae*.

Molecular and microbial annotations could assign clues about the lifestyles of each inhabitant of the office space. The majority of annotated molecules in the office space were traced back to the volunteer’s individual habits and daily routines. These results are consistent with our previous findings that the majority of molecules from lifestyles (i.e. personal care products, medications, food-derived molecules) in the chemical composition are derived from the human skin surface^12^ and that they can also be found on objects we touch^4^. Global Natural Products Social molecular networking^7^ provided level two annotations for some of the detected ions in this study, in accordance with the 2007 metabolomics standard initiative^13^. Relative mass spectrometry feature intensities and sub-Operational Taxonomic Unit (sOTU) abundances were further plotted in a recently introduced 3D cartography tool called ‘ili^10^ (Fig. 2a–p). Avobenzone (Fig. 2a), a major active ingredient in sunscreen, was found on many of the volunteers’ faces, hands, table, phones and computers, but was most intense on the computer, face and chest of volunteer 4. Diethyltoluamide (DEET) (Fig. 2b) was most abundant on the phone of volunteer 3 and also around the desk he was sitting next to. Laureth sulfate (Fig. 2c), a shampoo ingredient, was found on all volunteers as well as on the table. Amlodipine (Fig. 2d), also known as Norvasc™, is a calcium channel blocker used to treat high blood pressure, and was found primarily on the face and hands of volunteer 1 and the objects sitting in front of this person. A D-erythro-sphingosine (17:1) (Fig. 2e), a lipid produced by eukaryotes, was found in high abundance on the faces of all volunteers, and the flavonoid nobiletin (Fig. 2f) found in citrus was found in highest abundance on the computer and face of volunteer 4. The same molecule was also found in the lip balm of personal care product data set. Theophylline (Fig. 2g), a metabolism by product of caffeine, was found to be present on three of the volunteers although most intense on the face and hands of volunteers 1 and 2. Finally, azoxystrobin (Fig. 2h), is a known antifungal and previously described as a common food contaminant^14^ that is detected exclusively on the hand, computer and phone of volunteer 2.

Similarly, the spatial mapping of V4 16S rRNA amplicon data^15,16^ in the office space revealed many distinct localizations of specific bacterial communities. In total 44,393 unique sOTU were observed in this study. The soil bacteria *Nocardiaceae* (Fig. 2i), was abundant on the floor of the office. The environmental bacterium, *Acinetobacter guillouiae*, is often found in water, soil, and also feces^17^ (Fig. 2j). This bacterium was observed on the hands and face of volunteer 2. An unannotated species of Gram-negative nitrogen-fixing plant-associated^18^ ***α***-proteobacteria from the order *Rhizobiales* (Fig. 2k) was found on volunteer 1’s head, hands and upper back. A marine Cyanobacteria from the genus *Synechococcus* (Fig. 2I) was found on the body of volunteer 3. Actinomycetales (Fig. 2m) was found in high abundances on the body, phone and computer of volunteer 4. A bacteria from the genus *Staphylococcus* (Fig. 2n) was found to be specific to volunteer 2. A microbe associated with the human oral microbiome, *Veillonella parvula* (Fig. 2o), was found on the arms and chest of volunteer 1. Finally, *Chitinophagaceae* (Fig. 2p), a soil associated bacterium, was localized to the hands and phone of volunteer 3.

### Distinguishing environmental inhabitant “biome” uniqueness

**Figure 3:**
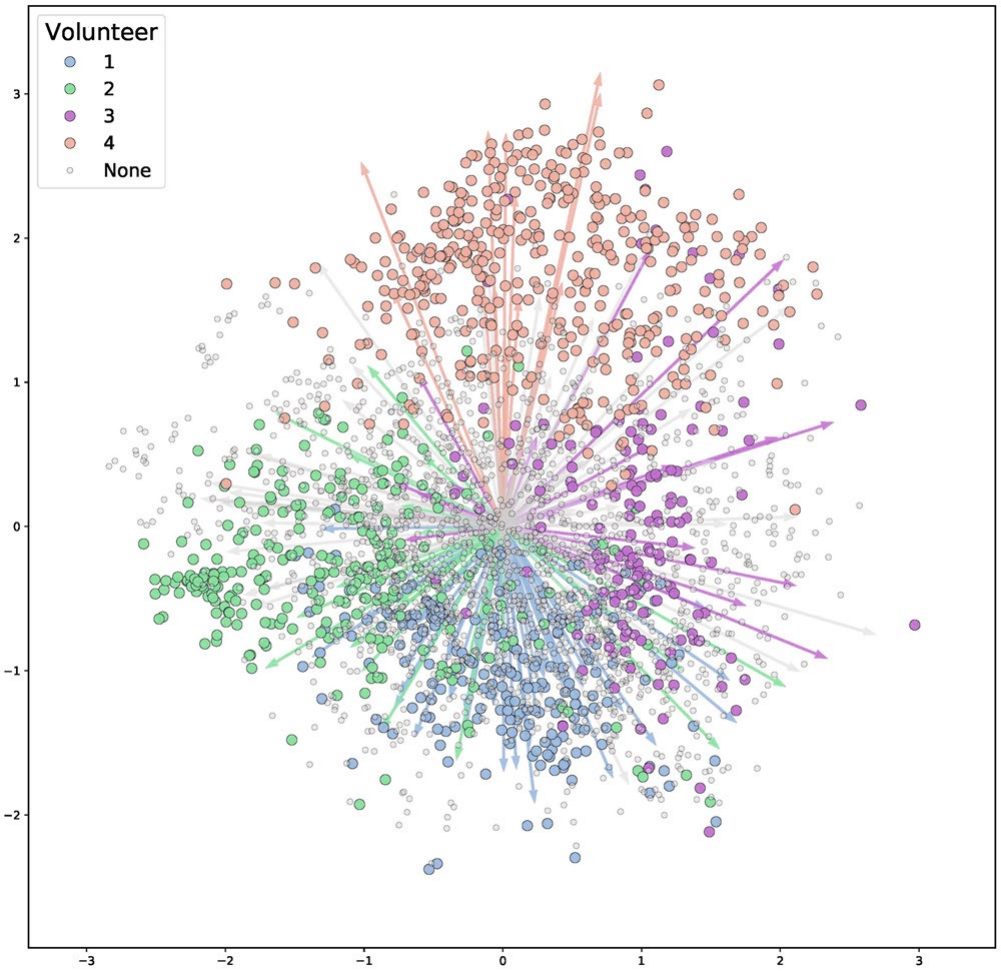
Partial least squares statistical biplot of volunteers and the built office space environment. Arrows represents unique microbes while circles represent unique molecules. Colors assign what unique volunteer the microbe or molecule was isolated from. Volunteer 1 is represented by the color blue, volunteer 2 is represented by color green, volunteer 3 is represented by color pink, and volunteer 4 is represented by color salmon. Color Grey illustrates microbes or arrows that cannot be correlated to a volunteer.

Once unique distributions of the molecules and microbes were established, a partial least squares^8^ (PLS) biplot was constructed to assess if the molecular “biomes” of each inhabitants were statistically distinguishable (Fig. 3). Partial least squares (PLS) was used for data representation as it was reported to perform well in the case of a low number of samples and a high number of dimensions (molecular features and microbial taxa)^8^. Molecular features are identified by inputting LC-MS/MS spectral data into the OpenMS^19^. Molecular features are defined as molecule’s atomic weight through mass to charge ratio (*m/z)* and retention time (RT). RT reflects the hydrophobicity property of the molecule. By combining these defining properties, molecular feature detection provides added confidence in distinguishing unique molecules from a complex mixture. Overlapping arrows (representative of microbes) and circles (representative of molecules) of a particular color illustrate that there is a correlation between the microbes and molecules within a volunteer. Volunteer 1 is represented by the color blue, volunteer 2 is represented by color green, volunteer 3 is represented by color pink, and volunteer 4 is represented by color salmon. Circles and arrows (microbes and molecules) that could not be matched to a volunteer are represented in gray. The overlapping of arrows and circles (microbes and molecules) of a single color demonstrate that there are unique correlations between the microbes and the metabolome of all volunteers. Furthermore, the top three molecular features in the PLS biplot were detected through OpenMS^19^ and microbial taxa through SortMeRNA^20^. Using this technique we were able to uniquely identify 41, 21, 23, 11 microbial reads that identify volunteers 1, 2, 3 and 4 respectively with a *p*-value less than 0.001 within this office environment. Additionally, we were able to characterize that 218, 255, 153, and 388 molecular features that uniquely identify each volunteer 1, 2, 3 and 4 respectively with *p*-value less than 0.001.

The top three microbes that had the highest PLS indicator value of 0.90, 0.83 and 0.79 for Volunteer 1 where a plant associated *Agrobacterium* bacteria, *Rhizobiales*, and *Rothia dentocariosa* oral cavity bacteria respectively (see Supplementary Table S1). The top three molecules that had the highest volunteer 1 PLS indicator value of 0.86, 0.81, and 0.78 were molecular features *m/z* 388.393 with a retention time (RT) range from 444 to 584 seconds (s), *m/z* 282.279, RT 448–583 s, and *m/z* 192.077, RT 193–203 s respectively (Supplementary Table 2). The top three microbes that had the highest volunteer 2 PLS indicator value of 0.91, 0.87 and 0.85 were *Staphylococcus* human skin bacteria, and two species of *Corynebacterium* (Supplementary Table 1). The top three molecules that had the highest volunteer 2 PLS indicator value of 0.84, 0.81, and 0.76 were molecular features *m/z* 374.326, RT 336–361, *m/z* 482.315, RT 388–413 s, and *m/z* 444.403, RT 402–445 s respectively (Supplementary Table 2). The top three microbes that had the highest volunteer 3 PLS indicator value of 0.94, 0.78, and 0.75 were two *Corynebacterium* and a match to whom the closest sequence was a *Deinococcus* extremophile respectively (Supplementary Table 1). The top three molecules that had the highest volunteer 3 PLS indicator value of 0.89, 0.87,and 0.86 were molecular features *m/z* 664.509, RT 577–601 s, *m/z* 669.466, RT 574–599 s, and *m/z* 655.995 RT 566–601 s respectively (Supplementary Table 2). The top three microbes that had the highest volunteer 4 indicator value of 0.82, 0.79, and 0.77 were the sequences that were most similar to *Deinococcus* bacteria, *Xanthomonadacae*, and *Corynebacterium* respectively (Supplementary Table 1). The top three molecules that had the highest volunteer 4 PLS indicator value of 0.96, 0.90,and 0.84 were unannotated molecular features *m/z* 368.424, RT 465–603 s, *m/z* 340.393, RT 444–599 s, and *m/z* 312.362, RT 410–575 s respectively (Supplementary Table 2). Additionally, seven microbes with statistically significant correlations to volunteer 3 (p-value < 0.001) are commonly found in marine environments (see Supplementary Table S3). These include marine species from the genera *Psychromonas, Marinomonas, Alteromonas, Vibrionaceae*, and, *Pseudoalteromonas* (Supplementary Table 1). Despite molecular annotation was limited across molecules with high correlations to specific volunteers, molecular feature *m/z* 311.164, RT 403–479 s identified as Avobenzone had a high correlation value to volunteer 4 at 0.80.

### Determining who touched what

**Figure 4:**
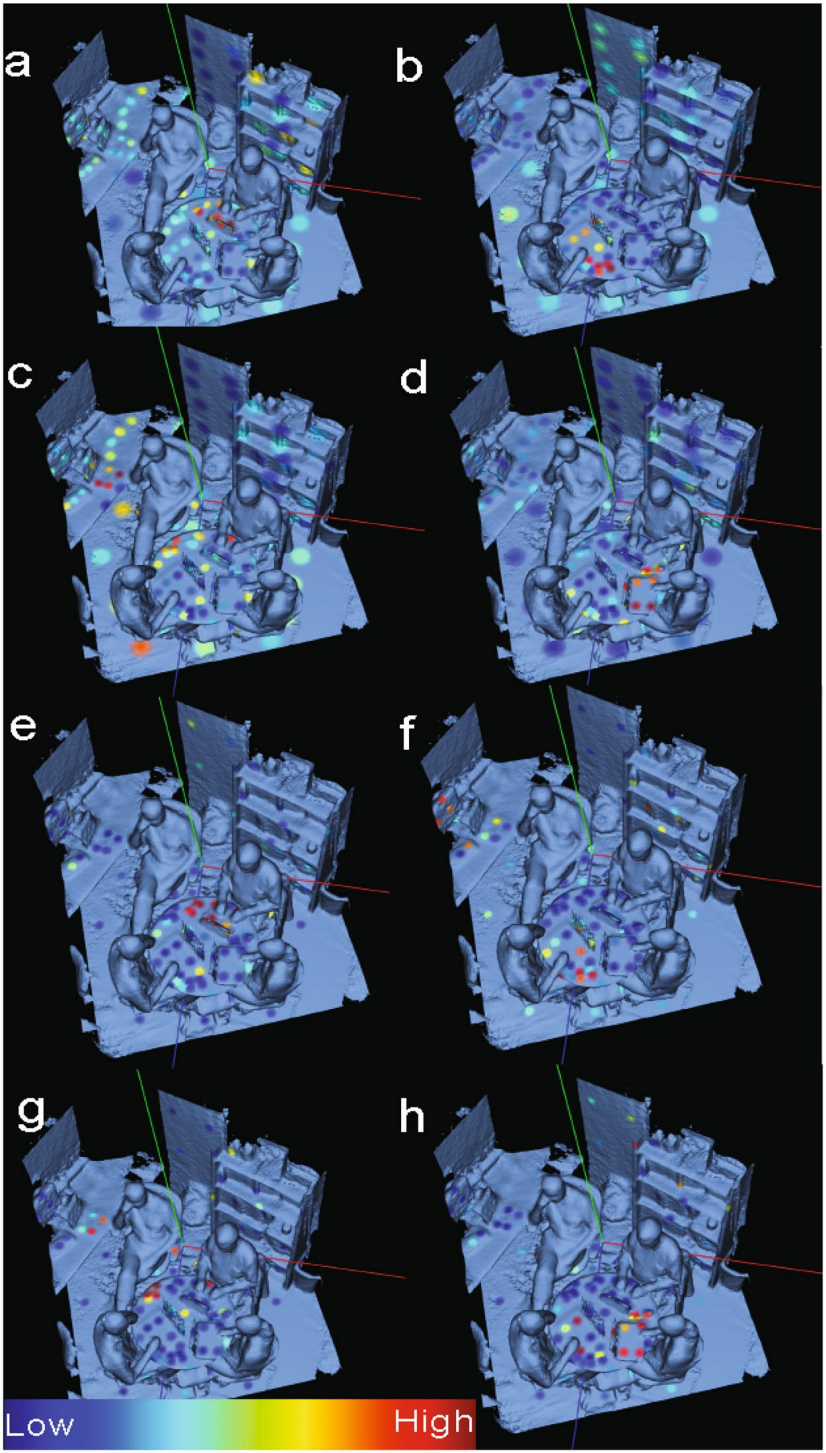
Distinguishing office interactions through chemical and microbial signatures using 3D cartography. Source tracking based on LC-MS profiling (**a**–**d**) as well as 16S amplicon microbial analysis (1e–h) illustrates the points and intensity of source tracking between volunteers 1–4 and samples sites across the office. Higher probability that molecular features from a sample site belong to a designated volunteer is indicated in red. Blue spots represent a lower probability that molecular features belong to a particular volunteer. Source tracking data based on volunteer 1 is shown in (**a,e**). Source tracking based on volunteer 2 is (**b,f**). Source tracking based on volunteer 3 is (**c,g**). Source tracking based on volunteer 4 are (**d,h**).

**Figure 5:**
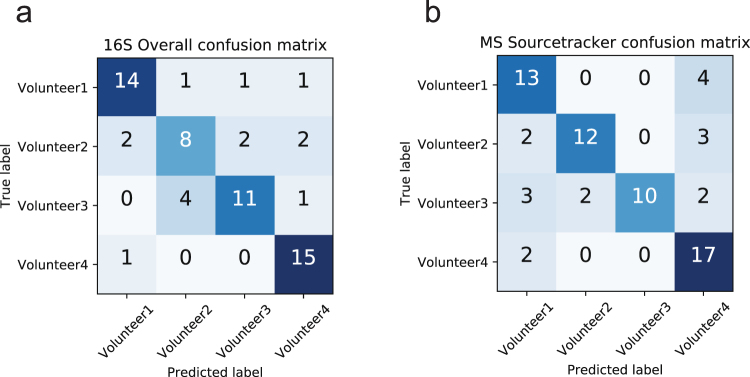
SourceTracker V.1 confusion matrices of metabolomic and microbial accuracy. (**a**) Using 16S input data in SourceTracker, 14 out of 17 total objects were correctly identified to belong to volunteer 1, 8 out of 14 objects were correctly identified to belong to volunteer 2, 11 out of 16 objects were correctly identified to belong to volunteer 3, and 15 out of 16 objects were correctly identified to belong to volunteer 4. (**b**) Using MS input data in SourceTracker, 13 out of 17 objects were correctly identified to belong to volunteer 1, 12 out of 17 objects were correctly identified to belong to volunteer 2, 10 out of 17 objects were correctly identified to belong to volunteer 3, and 17 out of 19 objects were correctly identified to belong to volunteer 4. Differences between total number of objects between volunteers were a result of each volunteer owned different amounts of objects. Differences between 16S and MS total amount of objects is a result of not having all of the complementary MS samples and 16S samples run experimentally.

In addition to individual molecules and microbes being mapped in 3D space and also each inhabitant’s “biome” possessing individual uniqueness, the interaction of inhabitants with their environments was also mapped (Fig. 4). SourceTracker^9^ is a Bayesian analysis that has previously been used to detect contamination in high-throughput metagenomic studies^17^. SourceTracker was used in this study to delineate the molecular interaction between each volunteer and the built environment. SourceTracker successfully correlated molecular profiles (MS accuracy = 0.742857142857, 16S accuracy = 0.761904761905) collected from the office space to those collected from participants (Fig. 5a,b). We show that chemistries from sample sites (table, desk and floor) within the office are highly correlated to the main inhabitant of the office space volunteer 3 (Fig. 4c,g). The chemical and microbial distribution of volunteers 1, 2, and 4 are limited to sites closer in spatial proximity to where the volunteers were physically sitting or objects they frequently interacted with that they brought into the room, i.e. phones, computers, and chair armrests (Fig. 4a,b,d–f,h).

## Discussion

Over the last decade, advances in mass spectrometry and DNA sequencing has made the technology cheaper and more accessible^21^. Although both have been widely used in forensics for some time^22–25^, it has yet to be used in the context of a global microbial and molecular inventory. Despite that microbial and untargeted metabolomics analysis for routine crime scene reconstruction forensics is still premature, modern microbial DNA and untargeted mass spectrometry technologies can provide many clues that would otherwise remain hidden from sight. These clues can be used in addition to existing forensic techniques in the reconstruction of a crime scene or potentially narrow down candidate suspects. Furthermore, this proof-of-concept experiment explored the value of combining molecular networking, PLS, Source tracking and 3D cartography and its potential application towards future forensics investigations.

Molecular networking not only revealed the origins of individual molecules, but also revealed an immense chemical overlap between inhabitants and the office space. Additionally, molecular networking was able to provide greater insight into where environmental molecules originate even if they didn’t match directly to an individual. As shown in Fig. 1f, nobiletin was found on the computer of volunteer 4, as well as the phones of volunteer 2 and 3. Since this molecule was not found on any individual, it was difficult to establish with confidence the ownership of this molecular feature. Figure 1g illustrates that although nobiletin was not matched specifically to an individual, it was matched to the lip balm personal care product of volunteer 4. This helped investigators hypothesize that the origin of the nobiletin is from volunteer 4 and was somehow shared to the personal devices of volunteer 2 and 3, which most likely occurred through direct contact. Without seeing direct overlap of chemicals form a person to a place, objects from suspects provided added evidence to establish origin of molecules. This illustrates the value in including non-human evidence or personal belongings that may be found outside of the investigation site. Discovering this relationship is important when identifying an individual’s chemical contribution to the surrounding environment.

More often than not, inhabitants of a place may not be present at the time of sample collection. Suspect formulation commonly rely on mugshot^26^ or fingerprinting^27^ while more modern approaches include technologies such as biometrics^28,29^, all which in isolation present clear ambiguity and biases^30,31^. This investigation supports the inclusion of lifestyle traces as a means to establish suspects within a given environment. As seen in Fig. 1, molecular annotation can be used to assign lifestyle clues of environmental inhabitants and can provide investigators with information of where inhabitants (or suspects) have been or where they may be going.

The widely distributed molecules like laureth sulfate (common household detergent) (1c), theophylline (caffeine derivative) (1g), avobenzone (sunscreen ingredient) (1a) (2D-erythro-sphingosine (eukaryotic lipid) (1d) may not be an effective molecular feature to formulate suspects in the office, but DEET (insect repellent) (1b) and Norvasc™ (medication) (1e) may be useful since they are specific to volunteers 3 and 1 respectively. These molecules are also found in close proximity in the built environment to these volunteers (Fig. 1) and may provide supplementary clues towards who is in the room and where they may frequent. Furthermore, molecules like azoxystrobin (common food contaminant pesticide^32^) found on the computer, hands, and phone of volunteer 2 illustrate that even molecules that are inadvertently encountered can serve as molecular signatures in trace levels. The microbial diversity and uniqueness found in the office space provides similar insights to the molecular annotations. It was expected to see soil and human bacteria (Fig. 1i–k,m–p) present in the room and unique to each individual. Surprisingly, marine bacteria unique to volunteer 3 (Fig. 1l) was an interesting supporting clue that separates this individual from the rest of the volunteers and also strongly suggests volunteer’s 3 interaction with the ocean. This could have significant implications when addressing who was present in a space, but as with any investigation, would require additional sampling from both environment and suspect.

A question that may arise when recreating a crime scene is to whether or not the human derived microbes or molecules are unique between each individual in the environment. In addition to demonstrating humans share a common space of molecular and microbial signatures, this investigation also supports that the biomes of each participant are unique and separate from each other at least in this particular instance in time. Although many molecules and microbes are shared across common space, PLS was able to illustrate that there is a subset of molecules and microbes that are unique to individuals (Fig. 2), which is supported by Indicator Value Analysis. As seen in Supplementary Table S2, the marine microbes identified on volunteer 3 may be useful as microbial fingerprints throughout the environment. Rather than attempting to identify possible suspects in a particular area based on their metabolome or microbiome, the uniqueness of each individual’s biome suggests their biome may be of value when determining their interaction with the surrounding environment. Although not yet directly demonstrated experimentally, we anticipate that PLS could be used in the future to help identify individuals to their microbes and molecules.

A key goal of this investigation was to understand how potential suspects interacted with the habitat and objects therein. Mapping SourceTracker in ‘ili illustrated interactions from both the microbe and molecular information (Fig. 3). Although SourceTracker accuracies hover at approximately ~75%, the successful correlation between person and objects at this scale successfully illustrate value in SourceTracker analysis. While volunteers (or potential suspects) 1, 2 and 4 interact on a molecular level in close proximity to each other. Volunteer 3 looks to interact more openly with the environment. Using primarily SourceTracker information, investigators concluded that volunteer 3 is the main inhabitant of the environment and much of the visiting inhabitants interaction is limited to their personal spaces or devices. This is reflected in the most significant associations of molecules and microbes to the floor, desk, table, and office phone. One can even gain insight into how this person uses the office as the molecules and microbes match best on the left side as this person enters the door to sit in the chair this volunteer is currently occupying. Additionally, the data suggests that volunteer 3 uses the right side of the office less frequently. SourceTracker also illustrated that much of the molecules and microbes belonging to volunteers 1, 2, and 4 are within close spatial proximity to the volunteers themselves. The localized distribution of these volunteer’s molecules and microbes reinforce the fact that they don’t interact with the environment often or at least as much as volunteer 3.

This study demonstrated an integrative approach of 3D cartography with mass spectrometric and microbial inventories to distinguish people within a group and also from their interactions with the built environment. Additionally, molecular and microbial annotations were used to predict behaviors and visited environments that individuals may experience outside of the investigated environment. Given the task to link, distinguish and predict how individuals interact with their environment, this approach offers alternative methods to traditional forensic techniques.

As with any investigation, scientific or judicial, controlling for error is of paramount importance. Although this proof-of-concept study provides novel insights into emerging molecular forensics capabilities, there are ethical, social and legal implications that surround such technologies. The scientific forensic community will have to tackle these societal and privacy challenges in the next few decades as the technology matures. Further developing the reliability and statistical accuracy of human-environmental interaction would greatly strengthen its merit in a courtroom, but in its current implementation it may help an investigator reconstruct what transpired in a room. It is important to note that the current state of this technology cannot and should not replace traditional forensics approaches. Additionally, the authors of this study hope that this proof-of-concept work will launch new research that will test temporal limits (how long do signatures last) and scalability (including multiple rooms, large number of suspects, etc.) of the approach introduced here.

## Methods

### Location selection

Office space is one of the most common human-occupied built environments in the United States, and was therefore selected as the primary site for this proof-of-principle study.

### Volunteers/IRB protocol #

Four volunteers were recruited to participate in this study. All Individuals signed a written informed consent and approved sample collection form their hands and personal objects in accordance with sampling procedures approved by the University of California San Diego Institutional Review Board protocol (IRB number **130537×**).

### Sampling

Volunteers were sampled 39 times across their body by taking individually pre-soaked EtOH/H20 cotton swabs and pressing to skin. Subsequently individually pre-soaked EtOH/H20 cotton swabs were pressed and rubbed against surfaces of the built environment. Each site on volunteer and sampling location was sampled twice for metabolite profiling and 16S microbial analysis. Sampling was performed at a 2 × 2 cm area of the head, forehead, cheek, nose, chin, bicep, forearm, hand, upper leg, lower leg, back, and bottom of shoe for each volunteer. Various 2 × 2 cm sample sites across the built-environment were also taken including chairs, shelves, desks, computers, mouses, cell phones, personal devices and phones. Pre-moistened cotton swabs were used at each site in 50:50 ethanol/water for MS analysis or 50 mM Tris pH 7.6, 1 mM EDTA, and 0.5% Tween 20 for nucleic acid analysis. Sample locations were noted, and swabs were placed in a deep-well 2-mL polypropylene 96-well micro titer plate.

### Extraction

DNA extraction is performed to capture microbial DNA from each sample that can be subjected to sequencing. The absorbed material was extracted in 500 μL of 50:50 ethanol/water (for mass spectrometric analysis) or Tris-EDTA buffer (50 mM Tris pH 7.6, 1 mM EDTA, and 0.5% Tween 20) for bacterial DNA extraction. After DNA extraction, amplification and sequencing according to the 16S rRNA amplicon protocol of the Earth Microbiome Project v.4.13 was performed^16^. DNA was extracted, amplified and sequenced at UC San Diego according to the 16S rRNA amplicon protocol of the Earth Microbiome Project v.4.13^16^.

### UPLC-MS/MS

At this stage of the methods, we separated molecules using chromatography and detected the ions of the molecules using a mass spectrometer. Processed office/volunteer extracts (5 uL) were subjected to UHPLC chromatographic separation using an UltiMate 3000 UHPLC system (Thermo Scientific), controlled by Chromeleon software (Thermo Scientific). Chromatographic separation was achieved using an 1.7 micron C18 (50 × 2.1 mm) Kinetex UHPLC column (Phenomenex) at 40 °C, using a flow rate of 0.5 mL/min. A linear gradient was used for the separation: 0–0.5 min 5% B, 0.5–8 min 5%B-99%B, 8–9 min 99%B, 9.01–10 min 1%B, 10–10.5 min 5–99% B, 11–11.5 min 99%B-1%B, 12–12.5 min 1% B where solvent A is water 0.1% formic acid (v/v) and solvent B is acetonitrile with 0.1% formic acid (v/v). Column eluent was introduced directly into a Bruker Daltonics maXis Impact quadrupole-time-of-flight mass spectrometer equipped with an Apollo II electrospray ionization source and controlled via otofControl v3.4 (build 16) and Hystar v3.2 software packages (Bruker Daltonics). The maXis instrument was first externally calibrated using ESI-L Low Concentration Tuning Mix (Agilent Technologies) prior to initiation of the sequence of samples, and hexakis (1H,1H,2H-difluoroethoxy)phosphazene (Synquest Laboratories), m/z 622.0295089613, was continuously introduced as an internal calibrant (lock mass) during the entirety of each LC/MS run. Data was collected in positive ion mode, scanning from 80–2000 m/z. Instrument source parameters were set as follows: nebulizer gas (Nitrogen) pressure, 2 Bar; Capillary voltage, 4,500 V; ion source temperature, 200 °C; dry gas flow, 9 L/min. MS1 spectral acquisition rate was set at 3 Hz and MS/MS acquisition rate was variable (5–10 Hz) depending on precursor intensity. Data-dependent MS/MS acquisition was programmed to the top five most intense precursors per MS^1^ scan and any precursor was actively excluded for 1 minute after being fragmented twice. Each MS/MS scan acquired was the average of 4 collision energies, paired optimally with specific collision RF (or “ion cooler RF”) voltages and transfer times in order to maximize the qualitative structural information from each precursor. The auto-acquisition of MS/MS spectra was carried according to specific settings. Precursor *m/z* 100, 300, 500, 1000 was selected; with an isolation width of 2, 4, 6, 8; with a base collision energy (eV) of 10, 25, 30, 50; a sampled collision energy of (5, 10, 15, 20), (12.5, 25, 37.5, 48), (15, 30, 45, 60), (25, 40, 75, 100); a collision RF (Vpp) of 250, 500, 1000, 1500 for all precursors; and a transfer time (μsec) of 50, 75, 100, 150 for all samples was used respectively. Data is publically available at http://gnps.ucsd.edu under accession number MSV000079181.

### Feature finding (MS data)

Feature finding is used to identify and distinguish detectible ions (molecules) from the complex sample mixture. Open-source OpenMS/TOPP software^19^ was used to identify molecular features from processed featureXML format. Unique features, represented by MS1 molecular mass, were identified based on detected precursor mass and retention time. An intensity table relating abundances of molecular feature per sample site was obtained automatically from samples. Feature detection parameters include: Threshold 0.95, *m/z* tolerance 10 ppm, and retention time tolerance 10 sec.

### Sequencing

DNA extraction and V4 paired end sequencing^15,16^ from samples were performed according to the Earth Microbiome Project Protocols^16^ and sequenced using an Illumina MiSeq (La Jolla, CA) to determine what bacteria are present at each sample site. Although V4 or any other amplification primer set has biases toward amplifying different organisms from humans^33–35^, including skin^36^, but also environmental microbes^16^, we and others have shown that V4 primers are sufficient to be used to trace them to people^36–40^ however as sequencing becomes cheaper performing the same task with metagenomes will increase the resolution with which the work outlined in this paper might be performed^41^. Processed tables can be found in https://qiita.ucsd.edu/study/description/10244, in addition sequences can be found in EBI under accession number ERP020615. Amplicon sequences were demultiplexed and quality controlled using the defaults as provided by QIIME 1.9.1. The primary sOTU table was generated using Qiita which uses deblurring^42^.

### QIIME

QIIME is an open-source bioinformatics pipeline for performing microbiome analysis from raw DNA sequencing data^43^. QIIME 1.9.1 was used to compute Hellinger distances on the MS and 16S datasets. The distance matrices were partitioned by sample type. Mantel tests were performed between the MS and 16S data controlling for the sample type. Principal coordinates were computed per sample type. Taxonomy of 16S sequences were assigned using the SortMeRNA method using Greengenes reference database.

### Microbiome/Metabolite correlations

Partial Least Squares (PLS) was used to correlate microbes and metabolites. This was performed using the PLSSVD function in scikit-learn, which calculates an SVD on the covariance matrix between log transformed metabolite and microbial abundances. A pseudocount was added to both datasets to avoid taking logs of zero. The loadings calculated from PLS were visualized using a biplot, where the points represent metabolites, and the arrows represent microbes. The angles between the arrow approximate the correlation between the corresponding microbes, and the distance between the points approximate the correlation between the corresponding metabolites^44^ package identify which microbes and metabolites are likely to be uniquely associated to specific volunteers. Points and arrows were colored by the volunteers that the corresponding metabolites and microbes were most likely associated with, with a p-value less than 0.001 and 1000 permutations.

### SourceTracker

A computational tool initially designed to predict sources and proportions of microbial contaminations in functional metagenomics studies was used. Bayesian statistics were calculated using SourceTracker V.1^9^ to determine the proportion of microbes and metabolites from the volunteers that were present in the objects. A confusion matrix was created to describe the performance of the SourceTracker classification model. True ownership of objects were known and tested by SourceTracker to achieve an accuracy value.

### GNPS dereplication

Global Natural Products Social Molecular Networking^7^ (http://gnps.ucsd.edu) dereplication was used to identify MS/MS spectral matches of molecular features in each sample set. Parameters for creating molecular network include a parent mass tolerance = 1.0 Da, Min matched Peak = 5, Ion Tolerance = 0.5, Score Threshold = 0.60 with default advance and filter search options (http://gnps.ucsd.edu/ProteoSAFe/status.jsp?task=0a0a825f41da4781a374cfb609283097). Using these parameters, 201,563 MS/MS spectra were merged into 17565 nodes. Raw data were uploaded to http://gnps.ucsd.edu and cataloged with a massive ID of MSV000079181.

### GNPS molecular networking

Global Natural Products Social Molecular Networking^7^ was used to identify MS/MS spectral matches of molecular features in each sample set. Parameters for creating molecular network include a parent mass tolerance = 0.1 Da, Min matched Peak = 6, Ion Tolerance = 0.5, Score Threshold = 0.80 with default advance and filter search options (http://gnps.ucsd.edu/ProteoSAFe/status.jsp?task=0a0a825f41da4781a374cfb609283097). Using these parameters, 301,113 MS/MS spectra were merged into 21,732 nodes. Raw data were uploaded to http://gnps.ucsd.edu and cataloged with a massive ID of MSV000079181. Networking parameters can be found with GNPS ID: http://gnps.ucsd.edu/ProteoSAFe/status.jsp?task=afed787019764b829ca7bf406500668f.

### 3D Cartography

Molecular feature visualization was performed to visualize microbiome and metabolome feautres on a 3D office model. The open-source web application ‘iliwas used by incorporating feature finding tables and STereoLithography (.stl) file (Supplementary material). For creating the 3D model, we used a 3D scanner (Structure bracket kit) attached to an iPad mini (Apple, Mountain View, CA, USA). Structure SDK application software was used to render and export the 3D model into the.stl file format.

### Data Availability

Analyses notebooks: https://github.com/knightlab-analyses/office-study. QIITA File: https://qiita.ucsd.edu/study/description/10244. Mass spectrometry is deposited in GNPS: Accession number MSV000079181. GNPS Molecular networking: http://gnps.ucsd.edu/ProteoSAFe/status.jsp?task=afed787019764b829ca7bf406500668f. Cartographical snapshot link for the 3D visualization of SourceTracker results for 16S: https://ili.embl.de/?ftp://massive.ucsd.edu/MSV000079181/updates/2017–08–16_ckapono_f9095c5e/other/3D%20office%20Source%20Tracker%20supplemental%20files/Model_wall%20(1).stl; ftp://massive.ucsd.edu/MSV000079181/updates/2017–08–16_ckapono_f9095c5e/other/3D%20office%20Source%20Tracker%20supplemental%20files/16S_ILI/sourcetracker_16s_ili_mapping2.csv; ftp://massive.ucsd.edu/MSV000079181/updates/2017–08–16_ckapono_f9095c5e/other/3D%20office%20Source%20Tracker%20supplemental%20files/16S_ILI/Volunteer%201.json. Cartographical snapshot link for the 3D visualization of SourceTracker results for MS: https://ili.embl.de/?ftp://massive.ucsd.edu/MSV000079181/updates/2017–08–16_ckapono_f9095c5e/other/3D%20office%20Source%20Tracker%20supplemental%20files/Model_wall%20(1).stl; ftp://massive.ucsd.edu/MSV000079181/updates/2017–08–16_ckapono_f9095c5e/other/3D%20office%20Source%20Tracker%20supplemental%20files/MS_ILI/sourcetracker_ms_ili_mapping.csv; ftp://massive.ucsd.edu/MSV000079181/updates/2017–08–16_ckapono_f9095c5e/other/3D%20office%20Source%20Tracker%20supplemental%20files/MS_ILI/Volunteer_1.json. Cartographical snapshot link for the 3D data of molecules (MS) and bacterial taxa (16S): https://ili.embl.de/?ftp://massive.ucsd.edu/MSV000079181/updates/2017–08–16_ckapono_f9095c5e/other/3D%20office%20Source%20Tracker%20supplemental%20files/Model_wall%20(1).stl; ftp://massive.ucsd.edu/MSV000079181/updates/2017–08–22_ckapono_11de23dd/other/ili_complete_fig_3.csv;ftp://massive.ucsd.edu/MSV000079181/updates/2017–08–16_ckapono_f9095c5e/other/3D%20office%20Source%20Tracker%20supplemental%20files/MS_ILI/Volunteer_1.json. Sequence data EBI accession number: ERP020615.

## Electronic supplementary material

Online Supporting Material
