# Supplementary material for: Creating a 3D microbial and chemical snapshot of a human habitat
Source: Sci Rep. 2018 Feb 27;8:3669. doi: 10.1038/s41598-018-21541-4 (PMC5829137; doi:10.1038/s41598-018-21541-4)
Supplement: Supplementary file 1 — Online Supporting Material [file 41598_2018_21541_MOESM1_ESM.doc]

**Online Supporting Material for: “Creating a 3D microbial and chemical snapshot of a human habitat”**

**Authors:** Clifford A. Kapono^1^, James T. Morton^3, 7^, Amina Bouslimani^2^, Alexey V. Melnik^2^, Kayla Orlinsky^3^ Tal Luzzatto Knaan^2^, Neha Garg^2^, Yoshiki Vázquez-Baeza^3^, Ivan Protsyuk^4^, Stefan Janssen^7^, Qiyun Zhu^7^, Theodore Alexandrov^2,4^, Larry Smarr^5,6^, Rob Knight^2,6,7^ *, Pieter C. Dorrestein^2,6^**

**Author Affiliations:**

1. Department of Chemistry, University of California San Diego, La Jolla, CA, USA

2. Collaborative Mass Spectrometry Innovation Center, Skaggs School of Pharmacy and Pharmaceutical Sciences, University of California at San Diego, La Jolla, CA, USA

3. Department of Computer of Science and Engineering, University of California San Diego, La Jolla, CA, USA

4. Structural and Computational Biology Unit, European Molecular Biology Laboratory, 69117 Heidelberg, Germany

5. California Institute for Telecommunications and Information Technology, University of California San Diego, La Jolla, CA, USA

6. Center for Microbiome Innovation, University of California San Diego, La Jolla, CA, USA

7. Department of Pediatrics, University of California San Diego, La Jolla, CA, USA

* To whom correspondence should be addressed regarding sequencing and statistics rknight@ucsd.edu ** to whom correspondence should be addressed regarding the project, 3D analysis and mass spectrometry pdorrestein@ucsd.edu.

In this online support material, we show (1) a 3D cartography map of the office environment and its inhabitants, (2) SortMeRNA^1^-assigned taxonomy microbiome correlations in respects to each inhabitant of the office space (3) GNPS^2^-assigned molecular annotation correlations in respects to each inhabitant of the office space and (4) SortMeRNA^1^-assigned taxonomy microbe correlations to volunteer 3.

1. Kopylova, Evguenia, Laurent Noé, and Hélène Touzet. "SortMeRNA: fast and accurate filtering of ribosomal RNAs in metatranscriptomic data." Bioinformatics 28.24 (2012): 3211-3217.

2. Wang, Mingxun, *et al*. "Sharing and community curation of mass spectrometry data with Global Natural Products Social Molecular Networking." *Nature Biotechnology* 34.8, 828-837 (2016).

**Supplementary Figure S1. 3D map of an office built environment.** Four occupants are sitting in chairs at a table with their personal computers and cellular phones. They are surrounded by a bookshelf, and the desk of volunteer 3.

**
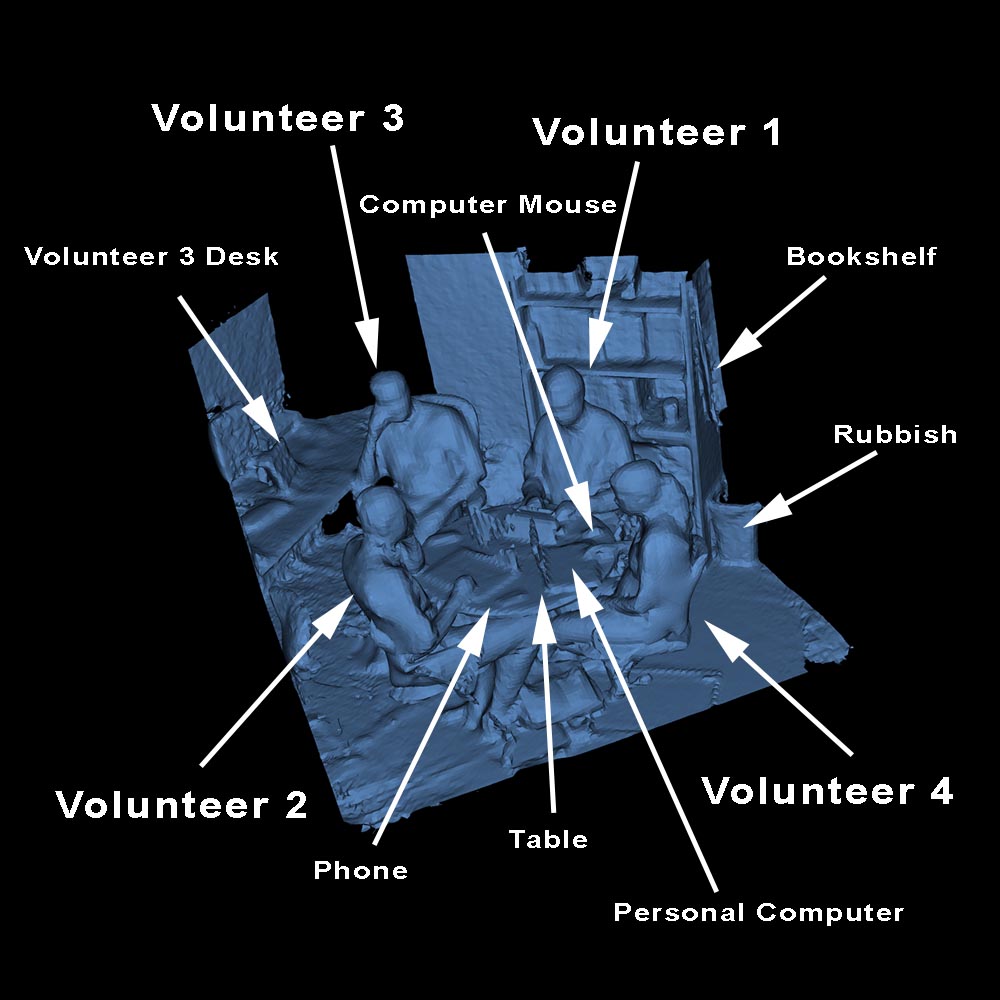
**

**Supplementary Table S1**. Microbiome Volunteer Correlations

| **Volunteer** | **Sequence** | **SortMeRNA-assigned taxonomy** | **Indicator value** | **p-value** |
| --- | --- | --- | --- | --- |
| 1 | TACGAAGGGGGCTAGCGTTGTTCGGAATTACTGGGCGTAAAGCGCACGTAGGCGGACATTTAAGTCAGGGGTGAAATCCCGGGGCTCAACCTCGGAACTG | *Agrobacterium* | 0.90 | 0.001 |
| 1 | TACGAAGGGGGCTAGCGTTGTTCGGAATCACTGGGCGTAAAGCGCACGTAGGCGGACCATTAAGTCAGGGGTGAAAGCCTGGAGCTCAACTCCAGAACTG | *Rhizobiales* | 0.83 | 0.001 |
| 1 | TACGTAGGGCGCGAGCGTTGTCCGGAATTATTGGGCGTAAAGAGCTTGTAGGCGGTTGGTCGCGTCTGCTGTGAAAGGCTGGGGCTTAACCCTGGTTTTG | *Rothia dentocariosa* | 0.79 | 0.001 |
| 2 | TACGTAGGTGGCAAGCGTTATCCGGAATTATTGGGCGTAAAGCGCGCGTAGGCGGTTTCTTAAGTCTGATGTGAAAGCCCACGGCTCAACCGTGGAGGGT | *Staphylococcus* | 0.91 | 0.001 |
| 2 | TACGTAGGGTGCGAGCGTTGTCCGGAATTACTGGGCGTAAAGAGCTCGTAGGTGGTTTGTCGCGTCGTCTGTGAAATCCCGGGGCTTAACTTCGGGCGTG | *Corynebacterium* | 0.87 | 0.001 |
| 2 | TACGTAGGGTGCGAGCGTTGTCCGGAATTACTGGGCGTAAAGAGCTCGTAGGTGGTTTGTCGCGTCGTTTGTGTAATACCGCAGCTTAACTGCGGGGTTG | *Corynebacterium* | 0.85 | 0.001 |
| 3 | TACGTAGGGTGCGAGCGTTGTCCGGAATTACTGGGCGTAAAGAGCTCGTAGGTGGTTTGTCGCGTCGTTTGTGGAATACCGCAGCTTAACTGTGGGGTTG | *Corynebacterium* | 0.94 | 0.001 |
| 3 | TACGAAGGTCCCAAGCGTTGTTCGGAATAACTGGGCGTAAAGCGTGTGTAGGCTGCGCGGAAAGTCAAATGTGAAAGCCAAGGGCTCAACCCTTGAACTG | *Verrucomicrobiaceae* | 0.78 | 0.001 |
| 3 | TACGGAGGGTGCGAGCGTTATCCGGAATCACTGGGCGTAAAGGGCGTGTAGGCGGGACGTTAAGTCTGGTTTTAAAGACCGCAGCTCAACTGCGGGAGTG | *Deinococcus* | 0.75 | 0.001 |
| 4 | TACGGAGGGTGCGAGCGTTATCCGGAATCACTGGGCGTAAAGGGCGTGTAGGCGGGACGTTAAGTCTGGTTTTAAAGACCGCAGCTCAACTGCGGGAGTG | *Deinococcus* | 0.82 | 0.001 |
| 4 | TACGAAGGGTGCAAGCGTTACTCGGAATTACTGGGCGTAAAGCGTGCGTAGGTGGTCGTTTAAGTCCGTTGTGAAAGCCCTGGGCTCAACCTGGGAACTG | *Xanthomonadaceae* | 0.79 | 0.001 |
| 4 | TACGTAGGGTGCGAGCGTTGTCCGGAATTACTGGGCGTAAAGAGCTCGTAGGTGGTTTGTCGCGTCGTTTGTGTAAGTCCACAGCTTAACTGTGGGACTG | *Corynebacterium* | 0.77 | 0.001 |

**Supplementary Table S2**. Metabolomics Volunteer Correlations

| **Volunteer** | **Molecular Weight (*m/z*)** | **Retention Time (sec)** | **GNPS Search Result** | **Indicator value** | **p-value** |
| --- | --- | --- | --- | --- | --- |
| 1 | 388.39 | 444-584 | No Matches | 0.86 | 0.001 |
| 1 | 282.27 | 448-583 | No Matches | 0.81 | 0.001 |
| 1 | 192.08 | 193-203 | No Matches | 0.78 | 0.001 |
| 2 | 374.33 | 336-361 | No Matches | 0.86 | 0.001 |
| 2 | 282.27 | 388-413 | No Matches | 0.81 | 0.001 |
| 2 | 192.08 | 402-445 | No Matches | 0.78 | 0.001 |
| 3 | 664.51 | 577-601 | No Matches | 0.89 | 0.001 |
| 3 | 669.46 | 574-599 | No Matches | 0.87 | 0.001 |
| 3 | 655.99 | 566-601 | No Matches | 0.86 | 0.001 |
| 4 | 368.42 | 465-603 | No Matches | 0.96 | 0.001 |
| 4 | 340.39 | 444-599 | No Matches | 0.90 | 0.001 |
| 4 | 312.35 | 410-575 | No Matches | 0.84 | 0.001 |

**Supplementary Table S3.** Marine Bacteria present on Volunteer 3

| **Sequence** | **Indicator value** | ***p*-value** | **SortMeRNA-assigned taxonomy** |
| --- | --- | --- | --- |
| TACGGAGGGTGCGAGCGTTAATCGGAATTACTGGGCGTAAAGCGCGTGTAGGTGGTTAATTAAGTCAGATGTGAAAGCCCAGGGCTCAACCCTGGAACTG | 0.181818182 | 0.001 | *Psychromonas* |
| TACAGAGGGTGCAAGCGTTAATCGGAATTACTGGGCGTAAAGCGCGCGTAGGTGGTTTGTTAAGTCTGATGTGAAATCCCAGGGCTCAACCTTGGAATGG | 0.267170702 | 0.001 | *Marinomonas* |
| TACGGAGGGTGCGAGCGTTAATCGGAATTACTGGGCGTAAAGCGCGCGTAGGTGGTTAGTTAAGTCAGATGTGAAATCCCAGGGCTCAACCTTGGAACTG | 0.27090713 | 0.001 | *Psychromonas* |
| TACGGAGGGTGCGAGCGTTAATCGGAATTACTGGGCGTAAAGCGCACGCAGGCGGTTTGTTAAGCTAGATGTGAAAGCCCCGGGCTCAACCTGGGACGGT | 0.333333333 | 0.001 | *Alteromonas* |
| TACGGAGGGTGCGAGCGTTAATCGGAATTACTGGGCGTAAAGCGCATGCAGGTGGTTCATTAAGTCAGATGTGAAAGCCCGGGGCTCAACCTCGGAACTG | 0.388894577 | 0.001 | Vibrionaceae |
| TACGGAGGGTGCGAGCGTTAATCGGAATTACTGGGCGTAAAGCGTACGCAGGCGGTTTGTTAAGCGAGATGTGAAAGCCCCGGGCTCAACCTGGGAACTG | 0.450119335 | 0.001 | *Pseudoalteromonas* |
| TACGGAGGGTGCGAGCGTTAATCGGAATTACTGGGCGTAAAGCGCGCGTAGGCGGTTAATTAAGTCAGATGTGAAATCCCAGGGCTCAACCTTGGAACTG | 0.555962764 | 0.001 | *Psychromonas* |
